# The burden of *Staphylococcus aureus* among Native Americans on the Navajo Nation

**DOI:** 10.1371/journal.pone.0213207

**Published:** 2019-03-05

**Authors:** Catherine G. Sutcliffe, Lindsay R. Grant, Angelina Reid, Grace K. Douglass, Robert C. Weatherholtz, Robin Hubler, Alvaro Quintana, Raymond Reid, Del Yazzie, Mathuram Santosham, Katherine L. O’Brien, Laura L. Hammitt

**Affiliations:** 1 Center for American Indian Health, Johns Hopkins Bloomberg School of Public Health, Baltimore, Maryland, United States of America; 2 Pfizer Vaccine Medical Development, Scientific and Clinical Affairs, Pfizer Inc, Collegeville, Pennsylvania, United States of America; 3 Navajo Epidemiology Center, Window Rock, Arizona, United States of America; Instiuto de Technologia Quimica e Biologica, PORTUGAL

## Abstract

**Introduction:**

Native Americans in the southwestern United States have a higher risk for many infectious diseases and may be at higher risk for *Staphylococcus aureus* due to the high prevalence of risk factors for *S*. *aureus*. Recent data on invasive *S*. *aureus* infections among Native Americans are limited.

**Methods:**

Active population- and laboratory-based surveillance was conducted in 2016–2017 on the Navajo Nation to document the rate of invasive *S*. *aureus*. A case of invasive *S*.*aureus* infection was defined as a Native American individual with *S*. *aureus* isolated from a normally sterile body site whose reported community of residence was on or around the Navajo Nation.

**Results:**

One hundred and fifty-nine cases of invasive *S*. *aureus* from 152 individuals were identified. The median age of cases was 56.3 years and 35% were female. Thirty-five percent of cases had community-acquired infections. Ninety-three percent of cases had underlying medical conditions, including diabetes (60%) and obesity (42%), 28% of cases had a documented prior *S*. *aureus* infection, and 33% were infected with methicillin-resistant *S*. *aureus*. The annual incidence of invasive *S*. *aureus* and of invasive methicillin-resistant *S*. *aureus* was 64.9/100,000 persons and 21.2/100,000 persons, respectively.

**Conclusions:**

This community has a high burden of invasive *S*. *aureus* infections. Further research is needed to identify prevention strategies and opportunities for intervention.

## Introduction

*Staphylococcus aureus* (SA) is a common cause of bacterial infections in the United States (US), causing non-invasive skin and soft tissue infections as well as invasive infections, including sepsis, pneumonia and necrotizing fasciitis. SA, particularly methicillin-resistant SA (MRSA), is responsible for a large number of hospitalizations and healthcare costs and is associated with significant all-cause mortality each year in the US [1–3]. In 2011, there were an estimated 80,461 cases and 11,285 deaths due to invasive MRSA in the US [1]. Historically, MRSA infections were acquired in healthcare settings, but the proportion of community-acquired infections attributable to MRSA increased in recent years [4].

Native American populations of the southwestern US experience higher morbidity and mortality from infectious diseases compared to the general US population [5–7], and may be at higher risk for SA due to the high prevalence of risk factors for SA [8], such as obesity, diabetes, and other health conditions [9]. From 1996 to 2005, the rate of MRSA-associated hospitalization, based on Indian Health Service (IHS) discharge data, among Native Americans in the southwestern US (including approximately 40% served by IHS facilities on Navajo Nation [10]) increased from 5.48 to 68.58 per 100,000 persons [11], significantly more than among Native Americans in other regions in the contiguous US [11]. The proportion of SA infections that are due to MRSA may also be higher among Native American communities than in the general US population [12]. While surveillance data indicate a decline in the rate of invasive MRSA infections in the general US in the last decade [1], recent data among Native Americans in the southwestern US are lacking.

This study assessed the epidemiology of invasive SA infections, including MRSA, among Native Americans on the Navajo Nation in the southwestern US.

## Materials and methods

### Ethics statement

This study was approved by the Navajo Nation, National Indian Health Service (IHS), and Johns Hopkins Bloomberg School of Public Health Institutional Review Boards. A HIPAA waiver was obtained to conduct medical chart reviews.

### Setting

This study was conducted on the Navajo Nation, the largest of the Native American tribal lands, with more than 200,000 tribal members, and lands covering more than 27,000 square miles in New Mexico, Arizona and Utah. Health care is provided free of charge to tribal members through the IHS or by tribally-administered facilities. Navajo Nation is located within the IHS Southwest region, which provides services to over 70 tribes in Arizona, New Mexico, Colorado, Utah and Nevada.

### Active bacterial surveillance

Active, laboratory and population-based surveillance for invasive SA began on May 1, 2016. The Center for American Indian Health (CAIH) at the Johns Hopkins Bloomberg School of Public Health has been conducting active surveillance for *Streptococcus pneumoniae*, *Haemophilus influenzae*, and *Neisseria meningitidis* on the Navajo Nation for over 20 years [6]. The protocol for the CAIH surveillance system is modeled on the Active Bacterial Core Surveillance system operated by the Centers for Disease Control and Prevention. Clinical microbiology laboratories at IHS and private facilities serving residents on Navajo Nation were contacted at least weekly to identify cases of invasive SA. We conducted monthly (IHS facility) and quarterly (private facilities) audits to identify additional cases that may have been missed. SA isolates were sent to the CAIH laboratory in Whiteriver, Arizona where they were confirmed using BBL CHROMagar Staph aureus plates (Franklin Lakes, NJ). For each case, a chart review was conducted to collect information on case demographics, underlying medical conditions, clinical syndrome, antimicrobial resistance test results, and health outcomes.

### Case definition

A case of invasive SA infection was defined as a Native American individual living in a community on or around the Navajo Nation with SA isolated from a normally sterile body site (e.g. blood, synovial fluid, deep tissue). Individuals may have contributed isolates from different body sources on the same day or isolates collected on different days. Individuals with isolates collected 7–29 days after the initial date of culture were considered persistent cases and all isolates were considered part of the same case event. For individuals with isolates from a specimen collected at least 30 days after the initial date of culture, the subsequent isolate defined a new case event and was defined as a recurrent case.

### Other definitions

**Fig 1:**
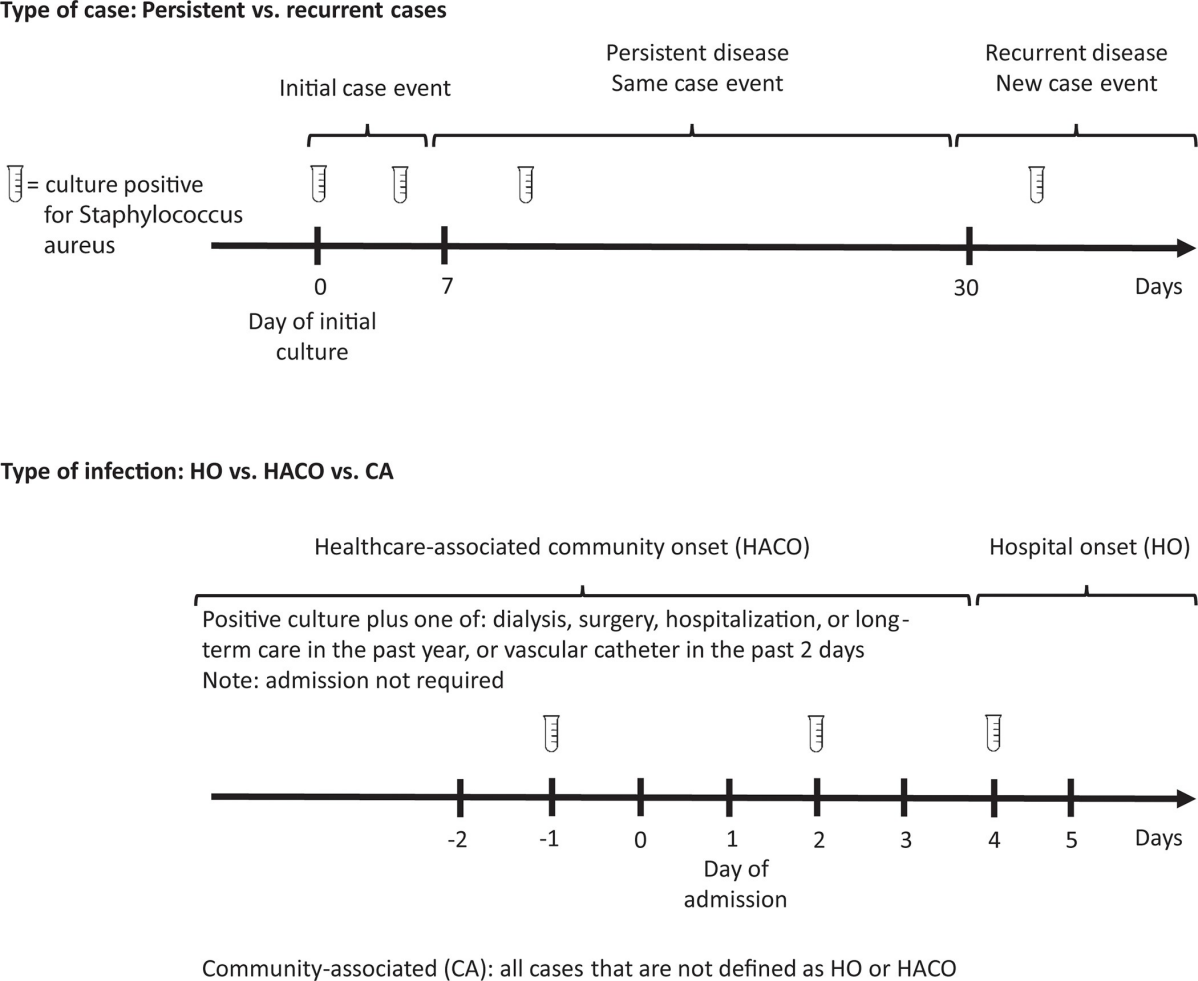
Definitions for the types of invasive *Staphylococcus aureus* cases and infections.

Infections were defined as hospital-onset (HO) when isolates were obtained from specimens collected >3 days after admission (with admission being day 1); as healthcare-associated community-onset (HACO) if cases had a healthcare risk factor (dialysis, surgery, hospitalization, or long-term care in the past year, or vascular catheter in the past 2 days) and isolates were obtained from specimens collected prior to or ≤3 days after hospital admission; or as community-associated (CA) for all other cases (Fig 1).

Disease syndromes associated with the invasive SA infection were defined based on the physician-reported syndrome in the medical record and the source of the isolate. Cases could have multiple syndromes reported.

Underlying medical conditions were documented based on medical record review (obesity additionally defined as present if body mass index ≥30). The Charlson Index, a measure of comorbidity, was calculated based on underlying conditions and age [13].

### Statistical analysis

Characteristics of cases were described and compared by infection type and antimicrobial resistance using chi-square tests for categorical variables and Wilcoxon rank sum tests for continuous variables.

Outcomes evaluated included hospitalization during the case event, amputation at or within 30 days after the initial culture, and death within 30 days after the initial culture. Outcomes were compared by infection type and antimicrobial resistance using chi-square tests.

Incidence rates of invasive SA were calculated using the IHS User Population from 2016 as the denominator. “Users” are defined as Native Americans receiving services at the IHS facility in the preceding 3 years [14]. Incidence was calculated overall, by age, and by antimicrobial resistance pattern using Poisson regression with robust variance estimation to account for recurrent infections. For comparison with the general US population [15], age-standardized incidence was calculated using direct standardization methods using the 2015 US Census Population as the reference group [16].

Analyses were performed using SAS statistical software, version 9.4 (SAS Institute Inc.) and Stata, version 14.2 (StataCorp).

## Results

From May 1, 2016 to April 30, 2017, 169 isolates of invasive SA were identified. They were contributed by 159 cases of invasive SA from 152 individuals (5 individuals had 1 recurrent infection; 1 individual had 2 recurrent infections during the study period).

### Characteristics of cases and isolates

The body sites of the 169 isolates were blood (n = 113; 66.9%), joint/synovial fluid (n = 25; 14.8%), bone (n = 15; 8.9%), deep tissue (n = 14; 8.3%), and peritoneal fluid (n = 2; 1.2%).

The median age of cases was 56.3 years (IQR: 42.8, 66.7; min, max: 1.5, 90.7) (Table 1). The majority of cases were male (n = 104; 65.4%). Healthcare exposures in the past year were common (Table 1). A majority of cases were defined as HACO (n = 93; 58.5%). For HACO cases hospitalized in the year prior to the case event (n = 80), the median time between the last discharge and the case event was 10.4 weeks (interquartile range [IQR]: 5.2, 23.4; min, max: 0.3, 52.0). For HO cases (n = 11; 6.9%), the median time between hospital admission and the date of invasive SA was 5 days (IQR: 4, 9; min, max: 4, 16).

**Table 1:** Demographic, healthcare and clinical characteristics of 159 cases of invasive *Staphylococcus aureus* disease, Navajo Nation, May 2016 –April 2017.

	N (%)
**Demographic characteristics**	
Male sex	104 (65.4)
Median age (IQR)	56.3 (42.8, 66.7)
BMI ^a^	
<25	44 (31.2)
25–29	44 (31.2)
≥30	53 (37.6)
Residence prior to culture	
Private residence	145 (91.2)
Long-term care facility	3 (1.9)
Hospital inpatient	3 (1.9)
Other	1 (0.6)
Unknown	7 (4.4)
**Healthcare exposures**	
Dialysis in past year	18 (11.3)
Surgery in past year	51 (32.1)
Hospitalized in past year	87 (54.7)
Long-term care in past year	16 (10.1)
Vascular catheter in past 2 days	8 (5.0)
**Underlying conditions: MRSA risk factors** ^**b**^	
Abscess/boil	16 (10.1)
Current smoker	12 (7.6)
Decubitis/pressure ulcer	18 (11.3)
Diabetes	96 (60.4)
Intravenous drug use	3 (1.9)
Malignancy	18 (11.3)
Obesity	66 (41.5)
**Underlying conditions: Other** ^**b**^	
Alcoholism	35 (22.0)
Chronic liver disease	19 (12.0)
Chronic pulmonary disease	1 (0.6)
Chronic renal insufficiency	26 (16.4)
Chronic skin breakdown	10 (6.3)
Congestive heart failure	18 (11.3)
Stroke	6 (3.8)
Dementia	11 (6.9)
HIV/AIDS	0
Myocardial infarction	4 (2.5)
Hypertension	36 (22.6)
Atherosclerosis / Peripheral vascular disease	21 (13.2)
Asthma	12 (7.6)
Other ^d^	26 (16.4)
**Disease syndrome(s)** ^**b**^	
Bloodstream infection (BSI) ^c^	113 (71.1)
Implant involved (Vascular catheter in past 2 days or other implant involved) ^d^	11 (9.7)
BSI with other focus ^d^	93 (82.3)
BSI without other focus	9 (8.0)
Pneumonia ^e^	25 (15.7)
Cellulitis or abscess ^f^	39 (24.5)
Osteomyelitis ^g^	36 (22.6)
Arthritis, joint infection, bursitis ^h^	34 (21.4)
Pericarditis or endocarditis ^i^	6 (3.8)
Urinary tract infection ^f^	9 (5.7)
Necrotizing fasciitis ^f^	4 (2.5)
Surgical site infection ^j^^,^ ^d^	19 (12.0)
Other	23 (14.5)
**Type of infection**	
CA	55 (34.6)
HACO	93 (58.5)
HO	11 (6.9)

BMI: body mass index; BSI: bloodstream infection; CA: community-associated; HACO: healthcare-associated community-onset; HIV/AIDS: human immunodeficiency virus/acquired immune deficiency syndrome; HO: hospital-onset; IQR: interquartile range; MRSA: methicillin-resistant *Staphylococcus aureus*

^a^ Among cases ≥18 years of age

^b^ Multiple categories per case are possible

^c^ Defined as SA isolated from blood

^d^ See S1 Table for more details

^e^ Defined based on reported diagnosis in medical record or SA isolated from pleural fluid

^f^ Defined based on reported diagnosis in medical record

^g^ Defined based on reported diagnosis in medical record or SA isolated from bone

^h^ Defined based on reported diagnosis in medical record or SA isolated from synovial fluid

^i^ Defined based on reported diagnosis in medical record or SA isolated from pericardial fluid

^j^ Defined by review of medical record if SA was isolated from the site of a prior surgical procedure occurring at any time before the date of culture

A quarter of cases (n = 44; 27.7%) had a documented prior SA infection, of whom 54.5% (n = 24) had a prior MRSA infection (S1 Table). Almost all cases had at least one documented underlying health condition (n = 148; 93.1%); the most prevalent conditions were diabetes (n = 96; 60.4%), obesity (n = 66; 41.5%), and alcoholism (n = 35; 22.0%; Table 1). The median Charlson Index was 3 (IQR: 1, 6; min, max: 0, 12). The most common clinical syndromes were cellulitis or abscesses (n = 39; 24.5%), osteomyelitis (n = 36; 22.6%), and arthritis, joint infection or bursitis (n = 34; 21.4%, Table 1). Nineteen cases (12.0%) had a surgical site infection from a surgery that occurred a median of 4.3 weeks (IQR: 2.1, 20.9) before the case event. A third (n = 52; 32.7%) of cases had MRSA.

Differences in the characteristics of invasive SA cases were found by infection type (S1 Table). HO cases were more likely to have cellulitis or an abscess or osteomyelitis and to have had a prior SA or MRSA infection. HO cases were also more likely to have an isolate that was methicillin resistant. HACO cases were more likely to have chronic renal insufficiency or asthma as an underlying condition, at least one underlying condition, a bloodstream infection, and a surgical site infection. CA cases were more likely to have arthritis or a joint infection. Differences were also found by antibiotic resistance profile (S1 Table). MRSA cases were more likely to be female, HO, and a resident of a long-term care facility in the past year. MRSA cases were also more likely to have had a prior MRSA infection, a malignancy, a history of asthma, and a bloodstream infection involving an implant.

### Outcomes among cases

The majority of cases were hospitalized (n = 136; 85.5%) during the case event. The median length of hospitalization was 11 days (IQR: 8, 21; min, max: 1, 63). Amputation of toes or feet was required for 17 (10.7%) cases; 9 (6.1%) cases died a median of 5 days (IQR: 3, 15; min, max: 1, 28) after the initial culture.

No differences in outcomes were found by antibiotic resistance (S1 Table). Differences by type of infection were found for the proportion of cases requiring hospitalization; CA cases were less likely to have been hospitalized during the case event.

### Annual incidence

The overall annual incidence of invasive SA was 64.9 per 100,000 persons (Table 2). For overall SA and MRSA, incidence increased with age and was highest among adults ≥65 years of age. By infection type, incidence of HACO infections was highest. The incidence of invasive MRSA was 21.2 per 100,000 persons. The age-adjusted incidence of invasive MRSA was 26.0 per 100,000 persons.

**Table 2:** Incidence of invasive *Staphylococcus aureus* disease by age, type of infection, and antibiotic resistance, Navajo Nation, May 2016 –April 2017.

	All SA cases N (%)	MRSA cases N (%)	Denominator	Incidence of SA per 100,000 persons (95% CI)	Incidence of MRSA per 100,000 persons (95% CI)
**Overall**	159 (100.0)	52 (32.7)	245135	64.9 (55.5, 75.8)	21.2 (16.2, 27.8)
**Age group (years)**					
<1	0	0	3753	0	0
1–17	6 (3.8)	2 (3.9)	70036	8.6 (3.9, 19.1)	2.9 (0.7, 11.4)
18–39	30 (18.9)	10 (19.2)	80382	37.3 (26.1, 53.4)	12.4 (6.7, 23.1)
40–49	21 (13.2)	3 (5.8)	26241	80.0 (52.2, 122.7)	11.4 (3.7, 35.5)
50–64	59 (37.1)	17 (32.7)	39362	149.9 (116.2, 193.4)	43.2 (26.9, 69.5)
≥65	43 (27.0)	20 (38.5)	25361	169.6 (125.8, 228.6)	78.9 (50.9, 122.2)
**Type of infection**					
CA	55 (34.6)	11 (21.2)	245135	22.4 (17.2, 29.2)	4.5 (2.5, 8.1)
HACO	93 (58.5)	32 (61.5)	245135	37.9 (31.0, 46.5)	13.1 (9.2, 18.5)
HO	11 (6.9)	9 (17.3)	245135	4.5 (2.5, 8.1)	3.7 (1.9, 7.1)
**Antibiotic resistance**					
MSSA	107 (67.3)	n/a	245135	43.6 (36.1, 52.8)	n/a
MRSA	52 (32.7)	n/a	245135	21.2 (16.2, 27.8)	n/a

N: number; n/a: not applicable; CI: confidence interval; CA: community-associated; HACO: healthcare-associated community-onset; HO: hospital-onset; MSSA: methicillin-susceptible *Staphylococcus aureus*, MRSA: methicillin-resistant *Staphylococcus aureus*, SA: *Staphylococcus aureus*

## Discussion

This study, which provides the first population- and laboratory-based estimates of invasive SA disease for the Navajo Nation, demonstrates a high burden of disease in this community. Most disease occurred among older adults and individuals with underlying medical conditions. Prior SA infections were reported for a quarter of cases, and recurrent infections were detected for four individuals in the 12-month study period, suggesting that exposure to SA in this community is common.

The observed age-adjusted incidence of MRSA (26.0 per 100,000 persons) was higher than that reported for the general US population in 2015 (18.8 per 100,000 persons) [15]. The incidence of invasive MRSA has declined in the US over the past decade, and the levels reported in this study are similar to that reported in 2011 in the general US population (25.8 per 100,000) [1]. The observed higher burden of MRSA on Navajo Nation is consistent with differences by race/ethnicity reported from Arizona: in 2017, the rates of invasive MRSA were 15.2 and 9.7 per 100,000 persons among Native Americans and White, non-Hispanic persons, respectively [17]. Few studies of the burden of overall SA or MRSA have been conducted among Native populations, thus limiting comparisons with other tribes in the region or in the US.

The age distribution of invasive MRSA cases in this study was similar to the general US population [1] and to MRSA cases among Alaska Natives [18], with the majority of cases occurring among older adults. This led to increasing incidence rates with age, which is also similar to the US general population [15] and the overall population in Arizona 2010 to 2015 [19]. Of note, the incidence rates of invasive MRSA in the older age groups on Navajo was much higher than those reported for Arizona in 2015 (approximately 30 and 50 per 100,000 for individuals 50–64 and ≥65 years, respectively) [19]. Although direct comparisons are limited by the different time period and surveillance methodologies, these results suggest there may be substantial intraregional variation.

Underlying health conditions (primarily diabetes, obesity, or alcoholism) and healthcare exposures were common among cases, both of which are commonly reported among MRSA cases in the US [1]. Only 34.6% of all invasive cases were classified as CA infections. The proportion of invasive MRSA cases classified as CA infections in this study (21.2%) was similar to what was observed in the general US population (20.7%) in 2015 [15].

The proportion of invasive cases that were MRSA in this study (32.7%) was lower than that observed in other studies in the US and Native American communities, although direct comparisons are complicated by differing case definitions across studies. In five large US hospitals, 42.9% of SA bacteremia isolates from 2008 to 2011 were MRSA [20]. In a Native American community in the Midwestern US, 55% of SA infections, including non-invasive infections, were MRSA [12], and at all IHS facilities in 2005, 52% of SA-associated hospitalizations were characterized as MRSA [11]. Reasons for a potentially lower proportion of MRSA in this population are unknown but could include differences in the time periods of the studies, patterns of antibiotic use within the IHS facilities on Navajo Nation or differing strains circulating within the communities. Unfortunately, no information is available on the prevalence of colonization with MSSA and MRSA on Navajo Nation. Additional molecular studies to understand the strains circulating in the community and causing invasive disease could help to understand differences between the epidemiology of SA on Navajo Nation and other regions in the US.

## Conclusions

The burden of invasive SA was high with cases occurring predominantly among older adults with underlying medical conditions. The incidence of invasive MRSA on Navajo Nation was higher than the general US population. This has important implications for prevention strategies, treatment algorithms, healthcare utilization and expenditure planning. Continued surveillance is necessary to establish trends in the incidence of invasive SA and MRSA disease over time. Additional research priorities include establishing community colonization prevalence and identifying risk factors for both colonization and invasive disease to guide prevention strategies.

## Supporting information

S1 Table Characteristics of cases of invasive *Staphylococcus aureus*, by antibiotic resistance and infection type, on Navajo Nation, May 2016 –April 2017. (DOCX)
