# Supplementary material for: The burden of Staphylococcus aureus among Native Americans on the Navajo Nation
Source: PLoS One. 2019 Mar 5;14(3):e0213207. doi: 10.1371/journal.pone.0213207 (PMC6400378; doi:10.1371/journal.pone.0213207)
Supplement: S1 Table — (DOCX) [file pone.0213207.s001.docx]

**Supplemental Table 1. Characteristics of cases of invasive *Staphylococcus aureus,* by antibiotic resistance and infection type, on Navajo Nation, May 2016 – April 2017**

| **DEMOGRAPHIC CHARACTERISTICS** | **Total**  **(N=159)^a^** | **By antibiotic resistance** | | | **By infection type** | | | |
| --- | --- | --- | --- | --- | --- | --- | --- | --- |
|  |  | **MSSA**  **(N=107)^a^** | **MRSA**  **(N=52)^a^** | **p-value^b^** | **HO**  **(n=11)^a^** | **HACO**  **(N=93)^a^** | **CA**  **(N=55)^a^** | **p-value^b^** |
| **Sex** |  |  |  | 0.01 |  |  |  | 0.72 |
| Male | 104 (65.4) | 77 (72.0) | 27 (51.9) |  | 6 (54.6) | 61 (65.6) | 37 (67.3) |  |
| Female | 56 (35.0) | 30 (28.0) | 25 (48.1) |  | 5 (45.5) | 32 (34.4) | 18 (32.7) |  |
| **Age group (years)** |  |  |  |  |  |  |  |  |
| Median (IQR) | 56.3 (42.8, 66.7) | 53.9 (42.1, 64.2) | 60.5 (43.9, 73.7) | 0.07 | 59.1 (33.6, 68.9) | 57.5 (43.6, 67.7) | 53.9 (39.2, 64.8) | 0.51 |
| <1 | 0 | 0 | 0 | 0.12 | 0 | 0 | 0 | 0.96 |
| 1-17 | 6 (3.8) | 4 (3.7) | 2 (3.9) |  | 1 (9.1) | 3 (3.2) | 2 (3.6) |  |
| 18-39 | 30 (18.9) | 20 (18.7) | 10 (19.2) |  | 2 (18.2) | 16 (17.2) | 12 (21.8) |  |
| 40-49 | 21 (13.2) | 18 (16.8) | 3 (5.8) |  | 1 (9.1) | 13 (14.0) | 7 (12.7) |  |
| 50-64 | 59 (37.1) | 42 (39.3) | 17 (32.7) |  | 4 (36.4) | 33 (35.5) | 22 (40.0) |  |
| ≥65 | 43 (27.0) | 23 (21.5) | 20 (38.5) |  | 3 (27.3) | 28 (30.1) | 12 (21.8) |  |
| **BMI^c^** |  |  |  | 0.27 |  |  |  | 0.40 |
| <25 | 44 (31.2) | 27 (28.4) | 17 (37.0) |  | 4 (40.0) | 26 (31.7) | 14 (28.6) |  |
| 25-29 | 43 (30.5) | 28 (29.5) | 16 (34.8) |  | 4 (40.0) | 20 (25.6) | 19 (38.8) |  |
| ≥30 | 54 (38.3) | 40 (42.1) | 13 (28.3) |  | 2 (20.0) | 35 (42.7) | 16 (32.7) |  |
| **Residence prior to culture** |  |  |  | 0.15 |  |  |  | 0.68 |
| Private residence | 145 (91.2) | 99 (92.5) | 46 (88.5) |  | 10 (90.9) | 85 (91.4) | 50 (90.9) |  |
| Long-term care facility | 3 (1.9) | 0 | 3 (5.8) |  | 0 | 3 (3.2) | 0 |  |
| Hospital inpatient | 3 (1.9) | 2 (1.9) | 1 (1.9) |  | 0 | 2 (2.2) | 1 (1.8) |  |
| Other | 1 (0.6) | 1 (0.9) | 0 |  | 0 | 1 (1.1) | 0 |  |
| Unknown | 7 (4.4) | 5 (4.7) | 2 (3.9) |  | 1 (9.1) | 2 (2.2) | 4 (7.3) |  |
| **CLINICAL CHARACTERISTICS** |  |  |  |  |  |  |  |  |
| **Type of infection** |  |  |  | 0.0003 |  |  |  |  |
| CA | 55 (34.6) | 44 (41.1) | 11 (21.2) |  | --- | --- | --- |  |
| HACO | 93 (58.5) | 61 (57.0) | 32 (61.5) |  | --- | --- | --- |  |
| HO | 11 (6.9) | 2 (1.9) | 9 (17.3) |  | --- | --- | --- |  |
| **Healthcare exposure** |  |  |  |  |  |  |  |  |
| Dialysis in past year | 18 (11.3) | 9 (8.4) | 9 (17.3) | 0.10 | 0 | 18 (19.4) | 0 | 0.0007 |
| Surgery in past year | 51 (32.1) | 34 (31.8) | 17 (32.7) | 0.91 | 5 (45.5) | 46 (49.5) | 0 | <0.0001 |
| Hospitalized in past year | 87 (54.7) | 53 (49.5) | 34 (65.4) | 0.06 | 7 (63.6) | 80 (86.0) | 0 | <0.0001 |
| Long-term care in past year | 16 (10.1) | 6 (5.6) | 10 (19.2) | 0.007 | 3 (27.3) | 13 (14.0) | 0 | 0.004 |
| Vascular catheter in past 2 days | 8 (5.0) | 3 (2.8) | 5 (9.6) | 0.07 | 0 | 8 (8.6) | 0 | 0.05 |
| **Prior SA infection** | 44 (27.7) | 27 (25.2) | 17 (32.7) | 0.32 | 7 (63.6) | 27 (29.0) | 10 (18.2) | 0.008 |
| **Prior MRSA infection** | 24 (15.1) | 9 (8.4) | 15 (28.9) | 0.0007 | 5 (45.5) | 12 (12.9) | 7 (12.7) | 0.01 |
| **Underlying conditions: MRSA risk factors^d^** |  |  |  |  |  |  |  |  |
| Abscess/boil | 16 (10.1) | 13 (12.2) | 3 (5.8) | 0.21 | 1 (9.1) | 10 (10.8) | 5 (9.1) | 0.94 |
| Current smoker | 12 (7.6) | 9 (8.4) | 3 (5.8) | 0.55 | 0 | 7 (7.5) | 5 (9.1) | 0.58 |
| Decubitis/pressure ulcer | 18 (11.3) | 10 (9.4) | 8 (15.4) | 0.26 | 1 (9.1) | 13 (14.0) | 4 (7.3) | 0.45 |
| Diabetes | 96 (60.4) | 65 (60.8) | 31 (59.6) | 0.89 | 9 (81.8) | 57 (61.3) | 30 (54.6) | 0.23 |
| Intravenous drug use | 3 (1.9) | 2 (1.9) | 1 (1.9) | 0.98 | 0 | 1 (1.1) | 2 (3.6) | 0.48 |
| Malignancy | 18 (11.3) | 8 (7.5) | 10 (19.2) | 0.03 | 0 | 14 (15.1) | 4 (7.3) | 0.17 |
| Obesity | 66 (41.5) | 48 (44.9) | 18 (34.6) | 0.22 | 3 (27.3) | 40 (43.0) | 23 (41.8) | 0.60 |
| **Underlying conditions: Other^d^** |  |  |  |  |  |  |  |  |
| Alcoholism | 35 (22.0) | 27 (25.2) | 8 (15.4) | 0.16 | 2 (18.2) | 21 (22.6) | 12 (21.8) | 0.95 |
| Chronic liver disease | 19 (12.0) | 15 (14.0) | 4 (7.7) | 0.25 | 0 | 13 (14.0) | 6 (10.9) | 0.38 |
| Chronic pulmonary disease | 1 (0.6) | 1 (0.9) | 0 | 0.48 | 0 | 0 | 1 (1.8) | 0.39 |
| Chronic renal insufficiency | 26 (16.4) | 15 (14.0) | 11 (21.2) | 0.25 | 0 | 22 (23.7) | 4 (7.3) | 0.01 |
| Chronic skin breakdown | 10 (6.3) | 7 (6.5) | 3 (5.8) | 0.85 | 2 (18.2) | 4 (4.3) | 4 (7.3) | 0.19 |
| Congestive heart failure | 18 (11.3) | 11 (10.3) | 7 (13.5) | 0.55 | 0 | 14 (15.1) | 4 (7.3) | 0.17 |
| Stroke | 6 (3.8) | 3 (2.8) | 3 (5.8) | 0.36 | 1 (9.1) | 3 (3.2) | 2 (3.6) | 0.63 |
| Dementia | 11 (6.9) | 5 (4.7) | 6 (11.5) | 0.11 | 1 (9.1) | 7 (7.5) | 3 (5.4) | 0.85 |
| HIV/AIDS | 0 | 0 | 0 | --- | 0 | 0 | 0 | --- |
| Myocardial infarction | 4 (2.5) | 3 (2.8) | 1 (1.9) | 0.74 | 0 | 3 (3.2) | 1 (1.8) | 0.75 |
| Hypertension | 36 (22.6) | 21 (19.6) | 15 (28.9) | 0.19 | 2 (18.2) | 21 (22.6) | 13 (23.6) | 0.92 |
| Atherosclerosis / Peripheral vascular disease | 21 (13.2) | 12 (11.2) | 9 (17.3) | 0.29 | 3 (27.3) | 15 (16.1) | 3 (5.4) | 0.06 |
| Asthma | 12 (7.6) | 4 (3.7) | 8 (15.4) | 0.009 | 0 | 11 (11.8) | 1 (1.8) | 0.05 |
| Other^e^ | 26 (16.4) | 15 (14.0) | 11 (21.2) | 0.25 | 3 (27.3) | 15 (16.1) | 8 (14.6) | 0.58 |
| **Any underlying condition** | 148 (93.1) | 99 (92.5) | 49 (94.2) | 0.69 | 10 (90.9) | 91 (97.9) | 47 (85.5) | 0.02 |
| **Charlson Index, median (range)** | 3 (0, 12) | 3 (0, 12) | 5 (0, 12) | 0.07 | 3 (0, 7) | 4 (0, 12) | 3 (0, 12) | 0.05 |
| **HOSPITALIZATION CHARACTERISTICS** |  |  |  |  |  |  |  |  |
| **Disease syndrome(s)^d^** |  |  |  |  |  |  |  |  |
| Bloodstream infection (BSI)^f^ | 113 (71.1) | 71 (66.4) | 42 (80.8) | 0.06 | 5 (45.5) | 75 (80.7) | 33 (60.0) | 0.004 |
| Implant involved (Venous catheter in past 2 days or other implant involved)^g^ | 11 (9.7) | 3 (4.2) | 8 (29.1) | 0.01 | 0 | 11 (14.7) | 0 | 0.06 |
| BSI with other focus^h^ | 93 (82.3) | 64 (90.1) | 29 (69.1) |  | 5 (100.0) | 56 (74.7) | 32 (97.0) |  |
| BSI without other focus | 9 (8.0) | 4 (5.6) | 5 (11.9) |  | 0 | 8 (10.7) | 1 (3.0) |  |
| Pneumonia^i^ | 25 (15.7) | 17 (15.9) | 8 (15.4) | 0.93 | 2 (18.2) | 15 (16.1) | 8 (14.6) | 0.94 |
| Cellulitis or abscess^j^ | 39 (24.5) | 26 (24.3) | 13 (25.0) | 0.92 | 6 (54.6) | 18 (19.4) | 15 (27.3) | 0.03 |
| Osteomyelitis^k^ | 36 (22.6) | 23 (21.5) | 13 (25.0) | 0.62 | 6 (54.6) | 17 (18.3) | 13 (23.6) | 0.02 |
| Arthritis, joint Infection, bursitis^l^ | 34 (21.4) | 27 (25.2) | 7 (13.5) | 0.09 | 1 (9.1) | 13 (14.0) | 20 (36.4) | 0.003 |
| Pericarditis or endocarditis^m^ | 6 (3.8) | 4 (3.7) | 2 (3.9) | 0.97 | 1 (9.1) | 5 (5.4) | 0 | 0.16 |
| Urinary tract infection^j^ | 9 (5.7) | 8 (7.5) | 1 (1.9) | 0.16 | 1 (9.1) | 3 (3.2) | 5 (9.0) | 0.29 |
| Necrotizing fasciitis | 4 (2.5) | 2 (1.9) | 2 (3.9) | 0.46 | 0 | 3 (3.2) | 1 (1.8) | 0.75 |
| Surgical site infection^n^ | 19 (12.0) | 13 (12.2) | 6 (11.6) | 0.91 | 0 | 18 (19.4) | 1 (1.8) | 0.003 |
| Other | 23 (14.5) | 19 (17.8) | 4 (7.7) | 0.09 | 1 (9.1) | 13 (14.0) | 9 (16.4) | 0.80 |
| **Antibiotic resistance** |  |  |  |  |  |  |  | 0.0003 |
| MSSA | 107 (67.3) | --- | --- |  | 2 (18.2) | 61 (65.6) | 44 (78.6) |  |
| MRSA | 52 (32.7) | --- | --- |  | 9 (81.8) | 32 (34.4) | 11 (20.0) |  |
| **Outcomes^d^** |  |  |  |  |  |  |  |  |
| Hospitalized during case event | 136 (85.5) | 88 (82.2) | 48 (92.3) | 0.09 | 11 (100.0) | 84 (90.3) | 41 (74.6) | 0.01 |
| Amputation | 17 (10.7) | 10 (9.4) | 7 (13.5) | 0.43 | 2 (18.2) | 9 (9.7) | 6 (10.9) | 0.69 |
| Death^o^ | 9 (6.1) | 5 (5.1) | 4 (8.0) | 0.49 | 1 (10.0) | 6 (6.8) | 2 (4.0) | 0.69 |

BMI: body mass index; BSI: bloodstream infection; CA: community-associated; HACO: healthcare-associated community-onset; HIV/AIDS: human immunodeficiency virus/acquired immune deficiency syndrome; HO: hospital-onset; IQR: interquartile range; MSSA: methicillin-susceptible *Staphylococcus aureus;* MRSA: methicillin-resistant *Staphylococcus aureus*; SA: *Staphylococcus aureus*

^a^ Numbers represent N (%) unless otherwise specified

^b^ p-value from chi-square test for categorical numbers and Wilcoxon rank sum test for continuous variables

^c^ Among cases ≥18 years of age

^d^ Multiple categories per case are possible

^e^ Includes 8 cases with hemiplegia/paraplegia, 7 with immunosuppression from steroids or other medications, 7 with cognitive deficit, 3 with burns, 2 with peptic ulcer disease, and 1 with transplant

^f^ Defined as SA isolated from blood

^g^ Includes 8 cases with vascular catheter, 1 with permacath, 1 with arteriovenous graft and 1 with pacemaker

^h^ Includes 26 cases with cellulitis/abscess, 23 with pneumonia, 13 with osteomyelitis, 14 with arthritis/joint infection/bursitis, 6 with endocarditis/pericarditis, 9 with urinary tract infection, 3 necrotizing fasciitis, and 19 with another syndrome listed. Cases could have multiple syndromes listed.

^i^ Defined based on reported diagnosis in medical record or SA isolated from pleural fluid

^j^ Defined based on reported diagnosis in medical record

^k^ Defined based on reported diagnosis in medical record or SA isolated from bone

^l^ Defined based on reported diagnosis in medical record or SA isolated from synovial fluid

^m^ Defined based on reported diagnosis in medical record or SA isolated from pericardial fluid

^n^ Defined by review of medical record if SA was isolated from the site of a prior surgical procedure occurring at any time before the date of culture. Includes 10 orthopedic surgeries on extremities, 3 spinal surgeries, 2 other orthopedic procedures (shoulder arthroscopy and steroid injection in hip), 2 catheter/port placements, 1 abdominal hernia repair with mesh, and 1 balloon-occluded retrograde transvenous obliteration.

^o^ Vital status unknown for 12 cases
